# Remineralizing Ability of Resin Modified Glass Ionomers (RMGICs): A Systematic Review

**DOI:** 10.3390/jfb14080421

**Published:** 2023-08-11

**Authors:** James Ghilotti, Paula Mayorga, José Luis Sanz, Leopoldo Forner, Carmen Llena

**Affiliations:** Department of Stomatology, Faculty of Medicine and Dentistry, Universitat de València, 46010 Valencia, Spain

**Keywords:** resin-modified glass ionomer cements, remineralization, dentin, in vitro

## Abstract

The selective caries removal approach leads to the need to use materials with the ability to remineralize remaining partially demineralized dentin. Among the materials proposed are resin-modified glass ionomer cements (RMGICs). The aim of this systematic review was to evaluate, based on in vitro experimental studies, whether RMGICs are suitable for remineralizing affected dentin. A systematic literature search was performed in four databases, followed by article selection, data extraction, and quality assessment. Studies assessing the remineralizing potential of RMGICs on dentin were included in our review. Studies which compared such properties between different RMGICs or with other materials were also eligible. The studies report the remineralizing ability of RMGICs, albeit with differences between different commercial products. RMGICs show a similar ability to conventional GICs to remineralize affected dentin, fulfilling the function for which they are designed. Moreover, the incorporation of additives, such as bioactive glass (BAG) or CCP-ACP, improves their remineralizing potential. The results of this review support the use of RMGICs as restorative materials after selective caries removal.

## 1. Introduction

Dentin is a highly mineralized organic tissue that, together with the dental pulp, forms the dentin–pulp complex [1]. In terms of its composition, it is majorly constituted of minerals (70%), organic components (20%), and water (10%) [2]. The mineral structure is formed by hydroxyapatite crystals arranged in different ways depending on the location. The organic components include type I collagen and phosphoproteins [2]. At a structural level, a distinction can be made between peritubular dentin, which is hypermineralized and has little collagen content, and intertubular dentin, which is formed by a network of collagen fibers with hydroxyapatite crystals arranged on their main axes [3].

Dental caries is a biofilm-mediated, diet-modulated, multifactorial, non-communicable, dynamic disease resulting in net mineral loss of dental hard tissues [4]. The microbial composition of cariogenic biofilms is not the same in enamel and dentin. Enamel is dominated by those of an acidogenic nature, while those with a proteolytic nature predominate on dentin [5].

In a healthy state, there is an equilibrium between the host and the microbial communities (eubiotic biofilm). However, under certain conditions, the interactions between the host and these microbial communities become unbalanced and the disease appears (dysbiotic biofilm) [6]. Overexposure to dietary carbohydrates is a factor which causes this imbalance of microbial communities and the transformation of a eubiotic biofilm into a dysbiotic biofilm. When the pH of the biofilm drops below the critical threshold (approximately 5.5), demineralization occurs. Under healthy conditions, the processes of demineralization and remineralization in enamel alternate in a dynamic equilibrium. When this equilibrium is broken, a net process of mineral loss occurs in the subsurface area of the enamel, giving rise to a weakened and porous enamel that corresponds to the initial caries lesion. If this situation can be reversed by increasing the periods of remineralization, mineral gain will occur. In this case, the caries lesion will stop or even remineralize [7]. Saliva is a protective factor against demineralization, primarily due to its dragging and cleansing effects and its mineral and organic composition that buffers pH changes and favors remineralization [8].

In the case of dentin, recovery is more complex than in enamel because it involves the reconstitution of two different phases: on the one hand, organic type I collagen and, on the other, inorganic apatite. This implies that remineralization alone is insufficient for the total recovery of demineralized dentin. In this manner, it is also necessary to restore the structure of the collagen matrix and for both phases to be linked in a specific way [9]. Biomineralization uses biomimetic analogs of dentin matrix proteins to induce amorphous calcium phosphate (ACP) nanoprecursors in the internal compartments of collagen fibers. This biomimetic remineralization process represents an approach based on creating nanocrystals that are small enough to fit into the gap zones between adjacent collagen molecules and establish a hierarchical order in the mineralized collagen [10]. A material capable of performing this process is considered bioactive.

Thus, the remineralization process in enamel is based on a gain of mineral components, whereas dentin remineralization involves a more complex process of interaction between mineral gain and its interaction with the collagen matrix.

Restorative dentistry aims to restore the functionality of dental tissues that have lost part of their structure due to dental caries. The longevity of these restorations is influenced by several factors, such as the considerable differences in the mechanical, physical, adhesive, and handling properties of the various restoration materials and adhesive systems [11]. A meta-analysis shows that posterior resin composite restorations have shown annual failure rates of 1 to 3%. The main causes of replacement are fractures and secondary caries [12]. To reduce the need for replacement due to secondary caries, materials with cariostatic properties have been developed [13].

The current minimally invasive treatment approach involves the removal of infected tissue, but it does include the preservation of affected tissue that is partially demineralized [14]. This requires the use of materials that have the ability to remineralize the preserved tissue. This process consists of restoring minerals through the formation of new inorganic mineral tissue [15]. Among the materials used for this purpose are glass ionomers.

Glass ionomers (GICs) are a group of materials whose polymerization is based on an acid–base reaction between the weak acid and the basic glass of which they are composed [16]. The calcium–fluor–alumino–silicate content in the glass powder is responsible for the remineralizing ability of GICs [17]. These materials are capable of releasing fluoride over long periods of time and even recharging with fluoride if it is present in high concentrations in the medium [18]. Fluoride release is considered beneficial because it promotes the formation of fluorapatite, which is slightly less soluble than hydroxyapatite [19]. When fluoride ions are released, they can saturate the liquid phase in and around the surface of the restorative tooth, resulting in the precipitation of CaF_2_ crystals. This reduces the chances of demineralization and accelerates the remineralization process. This process can be considered bioactive [11]. However, their mechanical characteristics are poor for many clinical uses [20].

Resin-modified glass ionomer cements (RMGICs) were developed to improve the physical and mechanical properties of GICs [21]. The most common resin monomer is 2-hydroxyethyl methacrylate (HEMA), to which a photoinitiator, such as camphoroquinone, is added to allow a light-mediated setting. These materials undergo a dual-setting reaction consisting of the typical acid–base reaction of GICs and photopolymerization [22]. However, the presence of resin monomers may affect the biological properties of RMGICs, especially with regard to their biocompatibility. This property decreases due to the cytotoxic effect of the resin component, especially during the first 24 h [23]. In order to improve the remineralizing potential of RMGICs, bioactive glasses are being incorporated into their composition, which also confers a bioinductive and regenerative potential to the material. Bioinductivity refers to the material’s ability to positively interact with living tissues by favoring cell migration and differentiation [24].

Given that the remineralization processes of enamel and dentin are different, as mentioned above, the present systematic review of in vitro studies is proposed with the aim of analyzing, based on the available scientific literature, the remineralizing potential of RMGICs on affected dentin and the possible positive effects of some of the additives they incorporate. These results could support the clinical use of these materials in deep caries lesions.

## 2. Materials and Methods

### 2.1. Protocol and Registration

This systematic review was performed following the PRISMA 2020 guidelines [25]. The protocol of this systematic review was previously registered in the Open Science Framework (OSF) registries (https://doi.org/10.17605/OSF.IO/SQ8VC (accessed on 3 February 2023)).

### 2.2. Inclusion and Exclusion Criteria

The research question of our review, based on the PICOS system, aimed to evaluate the current knowledge regarding the remineralizing potential of RMGICs on dentin, compared to a control or to another composition of RMGIC (P: resin-modified glass ionomers; I: application on demineralized dentin; C: comparison with other materials; O: dentin remineralization; S: in vitro studies).

In this way, in vitro studies evaluating the remineralizing potential of one or more RMGICs on dentin were eligible for inclusion in this review. The assessment of ion release from RMGICs was included among the assays on remineralization potential and thus was eligible for review.

Studies that only assessed cell adhesion on the materials were excluded as well as those that analyzed the materials’ cytotoxicity alone. Studies that assessed the remineralizing potential of RMGICs on dental enamel were also excluded.

### 2.3. Search Strategy

An advanced electronic search was performed on MEDLINE, Scopus, Web of Science, and Lilacs in October 2022.

The search strategy included the terms “resin-modified glass ionomer cement*”, “bioactivity”, “remineralization”, and “dentin”, combined with the Boolean operators AND and OR (Table 1). The selection of the search strategy was based on previous works within this framework and their most cited terms. In addition, the references of the included studies were manually screened after the selection process to verify additional potentially eligible studies.

The search results were exported from each database to a reference management software (Mendeley, Elsevier, Amsterdam, The Netherlands) to check for duplicates. Once the duplicates were removed, a first screening was performed of the titles and abstracts of the articles using the inclusion and exclusion criteria mentioned above. Studies that met the criteria were subsequently assessed for inclusion in the qualitative synthesis via full-text analysis.

### 2.4. Data Extraction

The data extraction process was subdivided into three separate categories: study characteristics, methodology, and results. Authors and years of publication were recorded as study characteristics. Methodological variables, in terms of the materials, included the following properties: the type of glass ionomers used and the material it was compared to. The variables related to remineralization assessment consisted of the following: the assay used and its duration. The outcome variables included the significant results found for each test, their significance value, and the time in which they were recorded (duration).

### 2.5. Quality Assessment

The checklist proposed by Faggion et al. [26] to evaluate the quality or risk of bias of in vitro studies on dental materials was used to assess the quality of the included studies.

## 3. Results

### 3.1. Study Selection and Flow Chart

**Figure 1:**
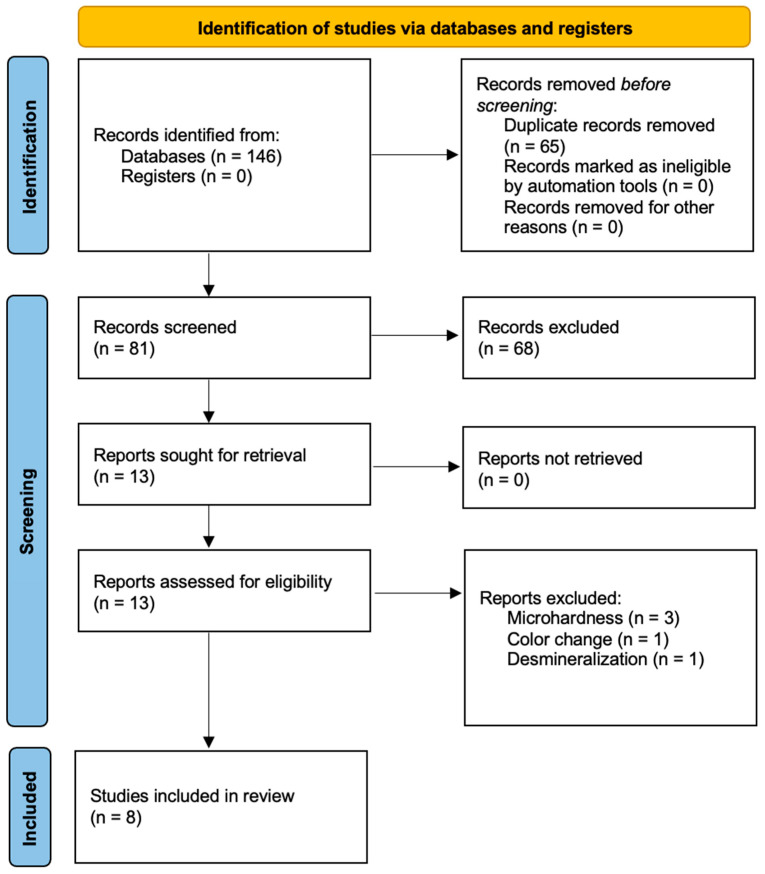
PRISMA flow diagram.

The search identified 146 references related to dentin remineralization by RMGICs, from which 54 were found in MEDLINE, 32 in Scopus, 54 in Web of Science, and 6 in Lilacs. After excluding 65 duplicates, 81 references remained to be examined. After reading the title and abstract, 68 references were excluded because they did not meet the inclusion criteria. Furthermore, after reading the full text of the 13 remaining articles, 8 were eligible for our review. Three were excluded because they assessed dentin microhardness, one was excluded for assessing dentin color changes, and another study was excluded for assessing demineralization (Figure 1). The selection of the articles was carried out in duplicate by two of the study researchers, JG and CL. In cases where there was no coincidence, the article was re-read jointly until a consensus was reached.

### 3.2. Quality Assessment

The quality of the included studies was assessed using the list proposed by Faggion [26] for the evaluation of in vitro studies on dental materials (Table 2). All of them had a correctly structured abstract (Item 1), as well as an introduction in which the scientific background, explanation of the rationale, and objectives are stated (Items 2 and 3). With the exception of the study by Xie et al. [27], the remaining studies explained the intervention performed in sufficient detail to allow replication (Item 3). In all cases, the outcome measures, both primary and secondary, were accurately defined, including how and when they were assessed (Item 4). No study stated how the sample size was determined nor was randomization performed (Items 5, 6, 7, 8, 9). The statistical methodologies used allowed for comparison by groups for primary and secondary outcomes (Item 10). Moreover, in six studies [27,28,29,30,31,32], for each outcome, the effect and precision were estimated with at least a 95% confidence interval (Item 11). The study’s limitations were only discussed in two cases [29,32], addressing sources of potential bias and imprecision (Item 12). The source of funding was stated in all but one of the included studies [29] (Item 13). Finally, none of the studies provided access to the full study protocol (Item 14).

### 3.3. Studied Materials

Different RMGICs and GICs were used in the studies (Table 3). Also, different additives were used with the intention of increasing the remineralizing effect as well as some experimental materials (Table 4).

### 3.4. Study Methodology and Results

The remineralization assays used by the included studies and their significant results are shown in Table 5.

In two cases [28,33], the assessment of remineralizing potential was performed by comparing the release of F^−^, Ca^2+^, and PO_4_^3−^ ions between the tested materials. The comparison between pure Vitro Fil LC (VFLC) and Resiglass F (RF) showed no differences, but when incorporating different percentages of bioactive glasses (BAGs) (45S5 and NbG), the release of ions increased in VFLC in all cases except for F^−^ in the VFLC + 5% 45S5 group. Contrarily, F^−^ release generally decreased upon BAG incorporation in RF, while Ca^2+^ increased and PO_4_^3−^ release exhibited a different behavior depending on the percentage of BAGs, i.e., decreasing at lower percentages (5%) and increasing at higher ones (20%). The addition of 30% of another BAG (S53P4) to Fuji II LC (FLC) also increased the release of F^−^, Ca^2+^, and SiO_4_^4−^ ions.

A wide variety of methods were used among the included studies to assess the remineralization potential of the tested materials. Scanning electron microscopy (SEM) showed a higher remineralization pattern of VFLC versus RF [28] in one study. In another study, it was used to confirm the remineralization ability of an experimental RMGIC [27]. It was also used to confirm that FLC + 30% S53P4 produces more calcified precipitates than without the incorporation of the BAG [33].

Fourier transform infrared spectroscopy (FTIR) was used in one study to assess remineralization potential [28]. A greater remineralization potential was observed from Ketac-Bond (KB) versus Vitrebond Plus (VP). However, in the same study, energy dispersive spectroscopy (EDX) results showed the opposite. Moreover, polarized light microscopy (PLM) showed greater remineralization when incorporating 10% BAG (S53P4) to Fuji II (FII) and FLC [29].

In two studies [30,31], transverse microradiography (TMR) was used to compare an experimental composite (ART Composite; AC) with FLC. At 2 weeks, in both cases, AC exhibited better results, while at 4 weeks, this occurred in only one of the studies. Finally, in one study [32], the analysis was carried out by micro-computed tomography (Micro-CT), and it was observed that by incorporating Casein phosphopeptide-amorphous calcium phosphate (CPP-ACP) to VII, greater remineralization was achieved.

## 4. Discussion

Currently, the conservative approach for the treatment of dental caries follows the criteria of the selective removal of carious dentin in combination with the use of materials with remineralizing, restorative, and/or bioinductive potential [35]. The European Society of Endodontology supports this approach and indicates the use of GICs as the indirect pulp-capping materials [14]. The remineralization process causes changes in the mineral structure of the dental hard tissue [36]. The need to place materials with remineralizing potential to recover the functionality of the affected dentin favored the implementation of GICs, and the need to improve their handling and mechanical properties led to the introduction of RMGICs. In contrast, it has already been demonstrated that the presence of resin in the composition can cause adverse effects on the pulp, such as inflammation. At the z clinical level, however, the results are positive even though they cannot be considered as biocompatible as traditional GICs [37].

There are already systematic reviews that point out the protective potential of RMGICs in the reduction of demineralization of the proximal dental tissue [38,39,40]. However, it is necessary to assess to what extent these materials can remineralize already affected dentin. Various randomized clinical trials showed that RMGICs have a high clinical efficacy in indirect pulp capping [41,42]. Accordingly, the aim of this study was to perform a systematic review of the available literature to assess dentin remineralization produced by these materials. Since this was a systematic review of in vitro studies, it was not possible to register it in PROSPERO.

Regarding the results, the assays used to assess the remineralization potential of RMGICs were very varied and did not follow a pre-established protocol [43]. This makes it difficult to compare studies in order to establish which materials best achieve this purpose. On several occasions, a traditional GIC was compared with an RMGIC. The most studied RMGIC was the FLC (in specific, five studies). Another aspect studied is the possible improvement of the remineralizing properties due to the incorporation of additives. In some cases, the incorporation of some additives showed promising results, such as CCP-ACP or BAG (45S5, NbG or S53P4), all of which resulted in a promotion of the remineralization. These additives had already demonstrated their capabilities in other studies independently [36,44,45,46]. The incorporation of these compounds into RMGICs has been shown to increase their remineralization potential [28,29,33].

Overall, the comparison between GICs and modified RMGICs has shown no significant differences, with small variations depending on the studies [34]. This result is of particular interest since, in studies assessing only fluoride release, it was concluded that the RMGICs had less remineralizing power. On evaluating remineralization by means of other more specific technologies, such as Micro-CT, SEM, FTIR, TMR, etc., it was shown that the incorporation of resin does not affect the materials’ remineralizing potential [47,48].

There are several articles that support the use of EDX to assess the remineralizing effect of GICs in association with FTIR [49,50,51], Micro-Raman [52], and XRD [53,54,55]. These techniques are less invasive and allow the analysis of the same sample at further endpoints as they do not require a coating. For this reason, the use of SEM–EDX is very useful for these studies and should be incorporated into the standard group of analyses performed to evaluated remineralization.

The new experimental compositions being studied should be taken into account for future research due to the positive results obtained. Materials such as EXP2.7(15) or AC have shown promising results with SEM and TMR compared to traditional RMGICs (FLC).

The mechanical properties of the studied materials were shown to be insufficient to withstand high masticatory loads. The incorporation of bioactive glasses may increase their microhardness, as shown in the study by Xie et al. [27]. However, in the study by Moraes et al. [28], no differences were observed. When RMGICs are used in areas of high masticatory load, it is appropriate to use the sandwich technique to reduce the risk of fracture of the restoration and the tooth substrate, showing similar results to those of a composite filling [56]. The higher mechanical strength, together with the fluoride release effect of RMGICs, reduces the possibility of secondary caries by forming fluorapatite, whose critical point of demineralization is lower than that of hydroxyapatite [57].

When assessing the quality of the included studies, a similar structural pattern is observed. Studies reported essential data, such as a sufficient abstract, clear objective(s), detailed description of the methodology, mention of statistical tests used, and relevant conclusions; but the sample size was never justified nor was randomization performed and, most importantly, study limitations were also not addressed in the discussion.

Current research directed towards the incorporation of additives to RMGICs requires the establishment of standardized protocols that allow their replication and a comparison between them. In this systematic review, only qualitative comparisons could be made due to the differences in the methodology of the included studies. This acts as a limitation of the present work. The main limitations of this study reside in the large variability of assays used to assess dentin remineralization, as well as the process to demineralize dentin, and the preservation conditions during the assays [58]. The use of standardized methodologies could allow for future reviews to perform a meta-analysis or quantitative synthesis. It should also be highlighted that the in vitro nature of the assessed studies’ results implies a limited application of the results of the present study to the clinical setting. Instead, the results from this review should be interpreted with caution and treated as preliminary evidence in this regard. Future studies are needed to confirm the reported results.

## 5. Conclusions

RMGICs show a similar ability to remineralize affected dentin as GICs in vitro, fulfilling the function for which they are designed. The incorporation of additives like BAG and CCP-ACP may potentially improve their remineralizing potential. Therefore, within the limitations of the in vitro nature of the included studies, this review supports the use of RMGICs as restoratives materials after the selective removal of dental caries.

## Figures and Tables

**Table 1:** Search strategy.

Database	Search Strategy	Findings
Medline	n° 1 (resin-modified glass ionomer cement*)	2003
	n° 2 (bioactivity) OR (remineralization)	154,091
	n° 3 (dentin)	42,496
	n° 1 AND n° 2 AND n° 3	54
Scopus	n° 1 (resin-modified glass ionomer cement*)	2142
	n° 2 (bioactivity) OR (remineralization)	88,229
	n° 3 (dentin)	44,380
	n° 1 AND n° 2 AND n° 3	32
Web Of Science	n° 1 (resin-modified glass ionomer cement*)	2376
	n° 2 (bioactivity) OR (remineralization)	94,721
	n° 3 (dentin)	52,709
	n° 1 AND n° 2 AND n° 3	54
Lilacs	n° 1 (resin-modified glass ionomer cement*)	277
	n° 2 (bioactivity) OR (remineralization)	626
	n° 3 (dentin)	8662
	n° 1 AND n° 2 AND n° 3	6

**Table 2:** Results of the assessment of in vitro studies by the use of the modified CONSORT checklist proposed by Faggion [26]. Cells marked with an asterisk “*” represent study fulfilment for the given quality assessment parameter. Cells left blank represent non-fulfilment.

Studies	Modified CONSORT Checklist Proposed by Faggion [17]														
	1	2a	2b	3	4	5	6	7	8	9	10	11	12	13	14
Xie et al. [27]	*	*	*		*						*	*		*	
Moraes et al. [28]	*	*	*	*	*						*	*		*	
Prabhakar et al. [29]	*	*	*	*	*						*	*	*		
Yang et al. [30]	*	*	*	*	*						*	*		*	
Zhang et al. [31]	*	*	*	*	*						*	*		*	
Zhao et al. [32]	*	*	*	*	*						*	*	*	*	
Yli-Urpo et al. [33]	*	*	*	*	*						*			*	
Toledano et al. [34]	*	*	*	*	*						*			*	

**Table 3:** List of RMGICs and GICs studied.

Material	Abbreviation	Composition	Manufacturer	Times Studied
Vitro Fil LC	VFLC	Powder: Fluorine silicate, strontium, aluminum, charge, activators, and iron oxide. Liquid: 2-hydroxyethyl methacrylate, aqueous solution of polyacrylic and tartaric acids, benzoyl peroxide, and camphorquinone.	DFL Indústria e Comércio, Rio de Janeiro, Brazil	1
Resiglass F	RF	Powder: Calcium fluorosilicate, barium, aluminum, polyacrylic acid, and inorganic fillers. Liquid: Dimethacrylate groups, deionized water, and catalysts.	Biodinâmica Química e Farmacêutica, Ibiporã, Paraná, Brazil	1
Fuji II	FII		GC Corporation, Tokyo, Japan	2
Fuji II LC	FLC	Powder: Fluro alumino silicate glass. Liquid: Distilled water, polyacrylic acid, 2-hydroxyethyl methacrylate, urethane dimethacrylate, camphorquinone.	GC Corporation, Tokyo, Japan	5
Ketac-Bond	KB	Powder: Calcium–aluminum–lanthanium–fluorosilica glass, pigments. Liquid: Polycarboxylic acid, tartaric acid, water, conservation agents.	3M Deustchland GmbH, Neuss, Germany	1
Vitrebond Plus	VP	Paste: HEMA, Bis-GMA, water, initiators, and radiopaque FAS (BL7AL). Liquid: Resin-modified polyalkenoic acid, HEMA, water, initiators.	3M Deustchland GmbH, Neuss, Germany	1
Experimental RMGIC	EXP	Powder: (Fluoro) alumino silicate glass. Liquid: light-curable star-shape poly(acrylic acid), water, 0.9% CQ, 1.8% DMAEMA, and 0.05% Hydroquinone.		1
Fuji VII	FVII	Powder: Fluro alumino silicate glass, Polyacrylic acid powder. Liquid: Polyacrylic acid, Polybasic carboxylic acid.	GC Corporation, Tokyo, Japan	1
Fuji VIII	FVIII	Powder: alumino silicate glass. Liquid: 2-HEMA 25–50%; tartaric acid 5–10%; 7,7,9(or 7,9,9)-trimethyl-4,13-dioxo-3,14-dioxa-5,12-diazahexadecane-1,16-diyl bismethacrylate 1–5%; 2-Hydroxy-1,3 dimethacryloxypropane 1–5%.	GC Corporation, Tokyo, Japan	1

**Table 4:** List of additives and other materials studied.

Material	Abreviation	Composition	Manufacturer	Times Studied
45S5	45S5	SiO_2_ = 45%, CaO = 24.5%, Na_2_O = 24.5%, P_2_O_5_ = 6%	Sylc, OSspray Ltd., London, United Kingdom	1
Niobophosphate glass (experimental)	NbG	Nb_2_O_5_ = 41.8%, P_2_O_5_ = 32.5%, CaO = 18.8%, Al_2_O_3_ = 2.7%, Na_2_O = 1.2%, SrO = 0.04%		1
S53P4 bioactive glass Frit	S53P4	SiO_2_ = 53%, Na_2_O = 23%, CaO = 20%, and P_2_O_5_ = 4%	MO-SCI^®^ Health Care, Rolla, MO, USA	1
Bioactive glass S53P4	S53P4	SiO_2_ = 53%, Na_2_O = 23%, CaO = 20%, and P_2_O_5_ = 4%	Vivoxid Ltd., Turku, Finland	2
ART Composite (experimental)	AC	Pyromellitic dianhydride glycerol dimethacrylate, Ethoxylated bisphenol A dimethacrylate, TTCP, silicon carbide/DCPA, DCPA, sodium hexaflurosilicate, Benzoyl peroxide, tertiary amine, camphorquinone		2
Casein phosphopeptide-amorphous calcium phosphate	CPP-ACP			1

**Table 5:** Summary of the results of included studies showing significant differences between different materials. *: *p* < 0.05, **: *p* < 0.01, ***: *p* < 0.001. SEM: Scanning electron microscopy; FTIR/ATR: Fourier transform infrared spectroscopy—attenuated total reflectance; PLM: polarized light microscopy; EDX: Energy-dispersive X-ray spectroscopy; FTIR: Fourier transform infrared spectroscopy; TMR: transverse microradiography; Micro-CT: micro computed tomography.

Studies	Materials	Assay	Time	Results
Xie et al. [27]	EXP2.7	SEM EDX	14 days	SEM: C - < EXP2.7(10), EXP2.7(15), EXP2.5(15) EDX: no difference
	EXP2.5			
	EXP2.7(10)			
	EXP2.7(15)			
	EXP2.7(20)			
	EXP2.5(10)			
	EXP2.5(15)			
	EXP2.5(20)			
Moraes et al. [28]	VFLC	Ion release F^−^	7 days	F^−^: VFLC + 5% 45S5 < VFLC < VFLC + 10% 45S5, VFLC + 20% 45S5, VFLC + 5% NbG, VFLC + 10% NbG, VFLC + 20% NbG *; RF + 5% 45S5, RF + 10% 45S5, RF + 20% 45S5, RF + 5% NbG, RF + 10% NbG < RF < RF + 20% NbG *
	VFLC + 5% 45S5			
	VFLC + 10% 45S5			
	VFLC + 20% 45S5			
	VFLC + 5% NbG	Ion release Ca^2+^		Ca^2+^: VFLC < VFLC + 5% 45S5, VFLC + 10% 45S5, VFLC + 20% 45S5, VFLC + 5% NbG, VFLC + 10% NbG, VFLC + 20% NbG *; RF + 5% 45S5, RF + 5% NbG < RF < RF + 10% 45S5, RF + 10% NbG, RF + 20% 45S5, RF + 20% NbG *
	VFLC + 10% NbG			
	VFLC + 20% NbG			
	RF			
	RF + 5% 45S5	Ion release PO_4_^3−^		PO_4_^3−^: VFLC < VFLC + 5% 45S5, VFLC + 10% 45S5, VFLC + 20% 45S5, VFLC + 5% NbG, VFLC + 10% NbG, VFLC + 20% NbG *; RF + 5% 45S5, RF + 5% NbG < RF, RF + 10% 45S5, RF + 10% NbG < RF + 20% 45S5, RF + 20% NbG *
	RF + 10% 45S5			
	RF + 20% 45S5			
	RF + 5% NbG			
	RF + 10% NbG	SEM	28 days	SEM: all VFLC < all RF
	RF + 20% NbG	FTIR/ATR		FTIR/ATR: no difference
Prabhakar et al. [29]	FII	PLM	28 days	FII < FII + 10% S53P4, FLC < FLC + 10% S53P4 **
	FII + 10% S53P4			
	FLC			
	FLC + 10% S53P4			
Yang et al. [30]	FLC	TMR	4 weeks	AC > FLC at 4 weeks *
	AC		8 weeks	AC = FLC at 8 weeks
Zhang et al. [31]	FLC	TMR	4 weeks	AC > FLC at 4 and 8 weeks *
	AC		8 weeks	
Zhao et al. [32]	FVII	Micro-CT	28 days	FVII + CPP-ACP > FVII, FVIII ***
	FVII + CPP-ACP			
	FVIII			
Yli-Urpo et al. [33]	FII	Ion release SiO_4_^4−^	1, 6, 24, 72, 168, 336 h	SiO_4_^4−^: FLC + 30% S53P4 > FII + 30% S53P4, FLC + 10% S53P4 > FII, FII + 10% S53P4, FLC at 72 h, 168 h; FLC + 30% S53P4 > all at 336 h ***
	FII + 10% S53P4			
	FII + 30% S53P4	Ion release F^−^		F^−^: FLC + 30% S53P4 > all at 72 h; FII + 30% S53P4 > all at 336 h
	FLC	Ion release Ca^2+^		Ca^2+^: FLC + 30% S53P4 > all at 168 h, 336 h
	FLC + 10% S53P4	Ion release PO_4_^3−^		PO_4_^3−^: FII + 30% S53P4 < all at 336 h
	FLC + 30% S53P4			
		SEM	336 h	FLC + 30% S53P4 has CaP precipitation on surface
Toledano et al. [34]	KB	SEM	336 h	FLC + 30% S53P4 has CaP precipitation on surface
	VP			

## Data Availability

The data presented in this study are available on request from the corresponding author.
